# The Complete Mitochondrial Genome of *Stichopus naso* (Aspidochirotida: Stichopodidae: *Stichopus*) and Its Phylogenetic Position

**DOI:** 10.3390/genes13050825

**Published:** 2022-05-05

**Authors:** Zhuobo Li, Bo Ma, Xiaomin Li, Ying Lv, Xiao Jiang, Chunhua Ren, Chaoqun Hu, Peng Luo

**Affiliations:** 1 CAS Key Laboratory of Tropical Marine Bio-Resources and Ecology (LMB), Guangdong Provincial Key Laboratory of Applied Marine Biology (LAMB), South China Sea Institute of Oceanology Chinese Academy of Sciences, Guangzhou 510301, China; lizhuobo19@mails.ucas.ac.cn (Z.L.); mabo20@mails.ucas.ac.cn (B.M.); lixiaomin19@mails.ucas.ac.cn (X.L.); jiangxiao@scsio.ac.cn (X.J.); rosemary166@sina.com (C.R.); hucq@scsio.ac.cn (C.H.); 2 University of Chinese Academy of Sciences, Beijing 100049, China; 3 Marin College, Beibu Gulf University, Qinzhou 535011, China; lvying0211@163.com; 4 Southern Marine Science and Engineering Guangdong Laboratory (Guangzhou), Guangzhou 510301, China

**Keywords:** Stichopodidae, *Stichopus naso*, mitogenome, phylogeny

## Abstract

The mitochondrial genome is widely used to study the molecular evolution of and perform phylogenetic analyses on animals. In this study, the complete mitochondrial genome (mitogenome) of *Stichopus naso* was sequenced. The mitogenome was 16,239 bp in length and contained 13 protein-coding genes (PCGs), 22 transfer RNA genes (tRNAs), and 2 ribosomal RNA genes (rRNAs). The genome composition showed positive AT-skew (0.023) and negative GC-skew (−0.158). The order of the mitochondrial genes was consistent with those from the *Stichopus* and *Isostichopus* species, whereas it was different from those of other species of Aspidochirotida. The phylogenetic analysis, based on the nucleotide sequences of 13 PCGs through the methods of Bayesian inference (BI) and maximum likelihood (ML), indicated that *S. naso* has close relationships with *S. horrens* and *S. monotuberculatus*, and belongs to a member of Stichopodidae. Our study provides a reference mitogenome for further molecular evolution studies and phylogenetic research on sea cucumbers.

## 1. Introduction

Mitochondrial DNA (mtDNA) is a type of extrachromosomal genome with unique characteristics, such as simple structure, low-rate recombination, maternal inheritance, and rapid evolution [1,2], and it is widely used as an effective molecular marker in diverse evolutionary research. Additionally, complete mitogenomes provide powerful tools for analyzing gene rearrangements and genomic evolution [3]. Therefore, mtDNA has been widely used in the study of genetic relationships and molecular identification, both within and among species of sea cucumbers [4,5]. The mitogenomes of most animals are typically closed circular molecules with a size of about 14-20 kb, including 13 protein-coding genes (PCGs), 22 transfer RNA genes (tRNAs), and 2 ribosomal RNA (12S rRNA and 16S rRNA) genes [2].

Sea cucumbers are an abundant and commercially important group of echinoderms. They inhabit a variety of marine environments, from intertidal zones to abyssal trenches [6,7]. Aspidochirotida is one of the most diverse orders in the Holothuroidea, including holothurians of various sizes [8], and the sea cucumbers in Aspidochirotida primarily occur in tropical waters featuring soft sediment and reefs [9]. Sea cucumbers play an important role in the benthic food web due to their deposit-feeding habit, acting as "vacuum cleaners" for coral reefs [10,11]. They are also available to humans as nutritious seafood with high economic value [12]. Although sea cucumbers have specific diversity, ecological importance, and high economic value, our understanding of their phylogenetic relationships and their evolution remains limited due to poor genomic and mtDNA data. Meanwhile, easily confused species (e.g. the members in the family Stichopodidae) cannot be accurately distinguished using traditional morphology, which has long been a taxonomic challenge [13]. Complete mitochondrial genomes provide abundant sequence information covering 13 PCGs, and they can be used for investigating phylogenetic relationships among species [14,15]. Among the 13 PCGs, 16S RNA and COX1 gene sequences are generally used for distinguishing confused species [16,17,18,19]. However, until now, only ~30 complete mitochondrial sequences of sea cucumbers have been released, which has seriously hampered the exploration of the phylogenetic relationships among sea cucumbers and other applications based on mitochondrial sequences.

*Stichopus naso* is a sea cucumber that is widely distributed in the Indo-Pacific region [20]. The sea cucumber is typically found in shallow water (1–19 m) on sandy muddy bottoms with or without sparse sea-grass beds [21]. The boundaries between species are not comprehensive or clear enough; therefore, *S. naso* is frequently misidentified as *S. horrens* [13]. In the present study, the complete mitogenome of *S. naso* was initially sequenced and compared with other mitogenomes of sea cucumbers. The genomic rearrangement and phylogeny were analyzed to deepen our understanding of the evolutionary relationships within the family Stichopodidae. The results of this research may provide new data for phylogenetic studies of the Aspidochirotida species and expand our knowledge about the mitochondrial genomic characteristics of sea cucumbers and the taxonomy within the family Stichopodidae.

## 2. Materials and Methods

### 2.1. Sample Collection and DNA Extraction

*Stichopus naso* was collected from Qionghai, Hainan Province, China. The body wall tissue was excised and preserved in 95% ethanol and stored at −20 °C until DNA extraction. Total genomic DNA was extracted using TIANamp Marine Animals DNA Kit (TianGen, Beijing, China).

### 2.2. Mitogenome Sequencing

The *S. naso* DNA library was sequenced by BIOZERON Co., Ltd. (Shanghai, China) using an Illumina NovaSeq 6000 with an average insert size of 450 bp, which was constructed using the NEBNext Ultra DNA Library Prep Kit. Approximately 5.11 GB of raw data from *S. naso* were generated with 150-base-pair paired-end read lengths. Adapters were removed and quality was trimmed with Trimmomatic v 0.39 [22]. Clean data was assembled de novo using SPAdes v3.14.1 (http://bioinf.spbau.ru/spades (accessed on 1 August 2021)) software with default parameters. Sequences with high coverage depth and long assembly length were selected as candidates and compared to NT libraries to confirm mitochondrial scaffold sequences, and sequences were ligated according to overlap. The starting position and orientation of the mitochondrial assembly sequences were determined from the reference genome of *Holothuria pervicax* (GenBank: MK328500.1) to obtain complete mitochondrial genome sequence of *S. naso*.

### 2.3. Sequence Annotation and Analysis

The complete mitogenome of *S. naso* was annotated using the online MITOS tool with the selection of echinoderm mitochondrial genetic code and BLAST searches in NCBI databases (https://blast.ncbi.nlm.nih.gov/Blast.cgi (accessed on 1 August 2021)) [23]. The base compositions and relative synonymous codon usage (RSCU) were conducted with MEGA 7.0 based on the echinoderm mitochondrial codon system [24]. Composition skew values were calculated according to the following formulas: AT- skew = (A − T)/(A + T); GC-skew = (G − C)/(G + C) [25]. The graphical map of the *S. naso* mitogenome was drawn using the CGview tool [26].

### 2.4. Phylogenetic Analysis

Phylogenetic analyses were performed using the sequences of 13 PCGs in mitochondrial genomes from 35 species, including sea cucumbers, sea stars, and sea urchins. In addition, three species of Echinoidea and three species of Asteroidea were included in the analyses as outgroups. Thirty-five mitogenomes were downloaded from GenBank (Supplementary Table S1) and the nucleotide sequences of their 13 PCGs were used to construct phylogenetic trees through maximum likelihood (ML) and Bayesian inference (BI) methods. Nucleotide sequences of the 13 PCGs were aligned with MUSCLE 3.8 [27]. The alignment results were further trimmed to eliminate the ambiguous positions using Gblocks v0.91 [28]. The trimmed sequences were concatenated into a supermatrix with FASconCAT [29]. Substitution saturation analysis was performed to detect the suitability of the dataset with 13 PCGs from all analyzed species for constructing a phylogenetic tree with DAMBE v. 5.3.15 [30]. The maximum likelihood (ML) and Bayesian inference (BI) were determined by using PhyML v3.0 [31] and MrBayes v3.2.6 [32], respectively. The best model was selected with jModeltest v2.1.10 [33] and the model of GTR + I + G was optimal for analysis with nucleotide alignment. Bayesian analysis was performed using four simultaneous Markov Chain Monte Carlo chains for 2,000,000 generations and sampled every 1000 generations, using a burn-in of 25% generations. The average standard deviation of split frequencies was <0.01. The resulting phylogenetic trees were visualized in FigTree v1.4.4 (http://tree.bio.ed.ac.uk/software/figtree/ (accessed on 22 November 2021)).

## 3. Results and Discussion

### 3.1. Genome Organization and Base Composition

**Figure 1:**
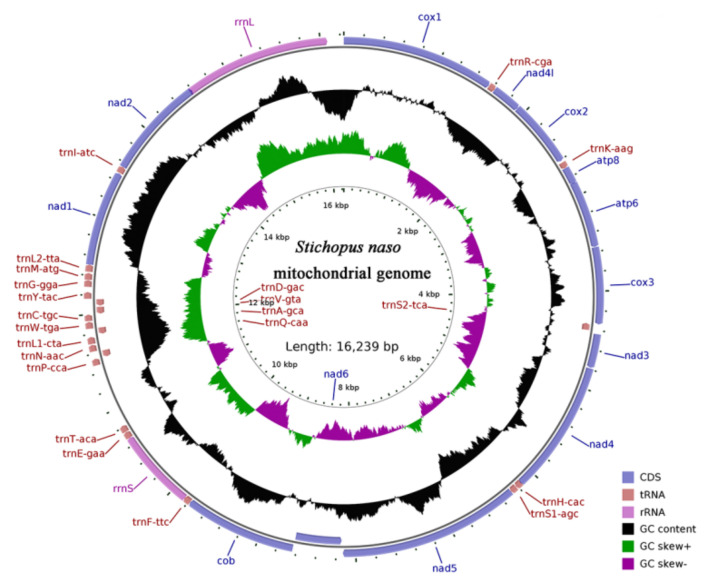
Gene maps of the *Stichopus naso* mitogenome. Blue arrows represent CDS, brown arrows represent tRNA, and lavender arrows represent rRNA, black, GC content, green, GC skew +, and dark purple, GC skew −.

The complete mitogenome of *S. naso* was acquired and deposited in GenBank under accession number MZ469138. The raw reads of the sequencing were deposited in the NCBI Sequence Read Archive (SRA Accession number: SRR19020101). The mtDNA of *S. naso* comprises 16,239 bp and includes 13 PCGs (*cox1–3*, *nad1–6*, *nad4L*, *cob*, *atp6*, and *atp8*), 22 tRNA genes, and 2 rRNA genes (*rrnS* and *rrnL*) (Figure 1) (Table 1). The order and orientation of the genes in the mitogenome are identical with those of other *Stichopus* species [34]. One of the thirteen PCGs (*nad6*) and five tRNAs (*trnS2*, *trnQ*, *trnA*, *trnV*, and *trnD*) are encoded on the light (−) strand, while the remaining 31 genes are located on the heavy (+) strand (Table 1). The base composition of the *S. naso* mitogenome is as follows: A = 4883 (30.07%), T = 4659 (28.69%), G = 2819 (17.36%), C = 3878 (23.88%) (Table 2). The A + T content of the mitogenome is 58.76%, which is lower than those of most of the sea cucumbers analyzed, but higher than those of *S. chloronotus* (58.55%), *Holothuria polii* (58.33%), and *H. leucospilota* (57.55%) (Supplementary Table S1) [35,36,37]. The bias toward high AT content is consistent with the general findings on the mitogenomes in metazoans [38]. The overall AT-skew and GC-skew in the entire *S. naso* mitogenome were 0.023 and −0.158, respectively. The AT-skew for the *S. naso* mitogenome was weakly positive, indicating a higher occurrence of A than T. By contrast, the GC-skew was slightly negative, indicating a higher incidence of C than G in the mitogenome. The potential mechanism leading to base composition bias in animal mitochondrial DNA may be related to transcription or replication [39]. The mutational stresses primarily associated with mitochondrial replication can have effects on the base composition of the mitochondrial genomes, which in turn creates base composition skews [40,41]. Thus, it suggests that these species may have been subjected to similar mutational pressures associated with mitochondrial replication. Both the base skew patterns of the *S. naso* were similar to those of other species of Aspidochirotida (Supplementary Table S1). The *S. naso* mitogenome possessed a 51-base-pair overlap between the genes in 7 locations ranging in size from 1 bp to 25 bp, and the longest overlap occurred between *nad2* and *rrnL* (Table 1). Three overlapping regions (*atp8*/*atp6*, *cox3*/*trnS2*, and *nad4*/*trnH*) were found in a variety of sea cucumbers [34,42,43,44,45].

### 3.2. Protein-Coding Genes and Codon Usage

The total length of the 13 PCGs in the *S. naso* mitogenome is 11,133 bp, which accounts for ~70.16% of the entire mitogenome. The average content of AT in the 13 PCGs is 58.07%. Moreover, the average AT-skew and GC-skew of the 13 PCGs are negative (Table 2). Most of the PCGs in *S. naso* begin with the standard ATG, except *nad3* and *nad2*, which initiate with the codon GTG. The start codon GTG is frequently found in genes encoding NADH dehydrogenase subunits (*nad1*, *nad4l* and *nad5*) and has also been observed in several sea cucumbers [43,44,45,46]. On the other hand, in the *S. naso* mitogenome, 10 PCGs (*cox1*, *nad4l*, *cox2*, *atp8*, *atp6*, *cox3*, *nad5*, *nad6*, *cob*, and *nad2*) end with TAA, while the remaining three PCGs (*nad3*, *nad4*, and *nad1*) terminate with TAG (Table 1). The usage of termination codons in the *S. naso* mitogenome is similar to those of other echinoderms [34,47,48].

**Figure 2:**
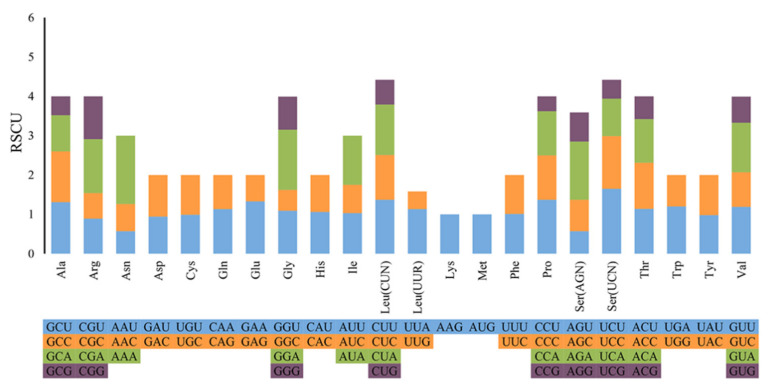
Relative synonymous codon usage in the *Stichopus naso* mitogenome. Codon families are indicated below the X-axis.

The relative synonymous codon usage (RSCU) values for the 13 PCGs are summarized in Table 3 and Figure 2. Excluding stop codons, there are 5252 codons in the mitogenome of *S. naso*. The codons encoding Leu (CUN), Asn, Ile, and Phe are the most frequent, while those encoding Cys and Met are scarce (Figure 2). These results were similar to those on the codon usage in the *S. horrens* mitogenome [34]. Moreover, AAA-Asn (RSCU = 1.74), is the most commonly used codon in the mitogenome of *S. naso*. The RSCU reflects the effect of biased codon usage [49]. In the mitogenome of *S. naso*, the bias toward greater representation of nucleotides A and T led to a corresponding bias in the encoding amino acids, and it is also a common feature of the mitochondrial genomes in most metazoans [15,50].

### 3.3. Transfer RNA and Ribosomal RNA Genes

**Figure 3:**
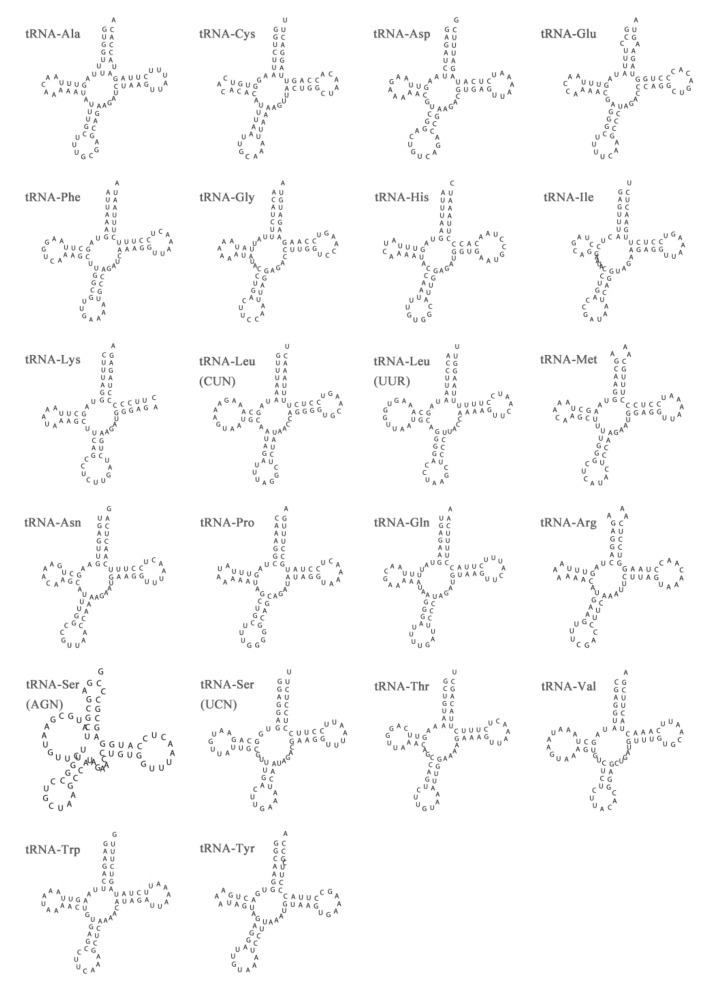
Putative secondary structures of tRNAs from the *Stichopus naso* mitogenome. The tRNAs are labeled with the abbreviations of their corresponding amino acids.

As with most Aspidochirotida mtDNAs, the *S. naso* mitogenome contains a set of 22 tRNA genes, ranging from 64 bp to 72 bp. The total length of the tRNAs in the *S. naso* mitogenome is 1518 bp, whereas the overall contents of AT and GC are 61.99% and 38.01%, respectively (Table 2). The tRNA genes of the mitogenome have a weakly positive AT skew (0.063) and a positive GC skew (0.043). The secondary cloverleaf structure of the 22 tRNAs was predicted (Figure 3). The *trnS* (AGN) lacked a dihydrouridine (DHU) arm, which is considered a common phenomenon in metazoan mitogenomes [51,52,53,54,55]. Except for *trnS*, the other tRNA genes exhibit typical cloverleaf structures. Previous studies on metazoans have shown that truncated tRNAs lack the D-arm without losing their function [56,57]. Except for *trnR*, 32 non-Watson–Crick G-U were recognized in the other tRNAs of the *S. naso* mitogenome. G-U base pairs were also found in *Benthodytes marianensis* and *Phyllophorus liuwutiensis* [42,44]. Previous studies have shown that mismatches can be corrected by the editing process without impacting the tRNA transport function [58].

As in other sea cucumbers, the *rrnS* and *rrnL* genes generally locate between *trnF* and *trnE*, and between *nad2* and *cox1*, respectively (Table 1). The lengths of the *rrnS* and *rrnL* genes are 827 bp and 1496 bp, respectively. The A + T content of the rRNAs is 58.37%, which is lower than that of other mitogenomes from the *Stichopus* species (Supplementary Table S1). Nevertheless, the average skewed values of AT and GC for the two rRNAs are positive (0.203) and negative (−0.036), respectively, which is similar to other *Stichopus* species (Supplementary Table S1).

### 3.4. Gene Rearrangement

**Figure 4:**
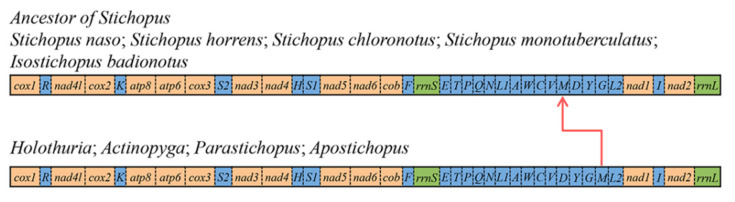
Linear representation of gene rearrangements of *Stichopus naso*, *S. horrens*, *S. chloronotus*, *S. monotuberculatus*, *Isostichopus badionotus*, *Holothuria*, *Actinopyga*, *Parastichopus*, and *Apostichopus*. All the genes are transcribed from left to right. The tRNA genes are represented by the corresponding single-letter amino acid code. S1 (AGN), S2 (UCN), L1 (CUN), and L2 (UUR). The *rrnL* and *rrnS* are the large and small ribosomal RNA subunits, respectively. The red arrow represents the transposition event, which shifts *trnM* from the position between *trnG* and *trnL2* to the position between *trnV* and *trnD*.

Mitochondrial gene rearrangement can be an effective tool to study phylogenetic relationships [59,60]. In order to detect the occurrence of rearrangements in *S. naso*, the gene orders of *S. naso* and other Aspidochirotida species were compared (Figure 4). A transposition event was observed in the *S. naso* mtDNA, and it shifted *trnM* from the site between *trnG* and *trnL2* to the site between *trnV* and *trnD* (Figure 4). In a manner that was consistent with previous reports, the tRNA genes appeared to be among the most active elements in the mitochondrial genomes [34,57], and the rearrangement of tRNA genes may be achieved by replication or intramolecular translocation [57]. The tandem duplication/random-loss model (TDRL) is one of the most widely adopted mechanisms for mitochondrial gene rearrangement, which assumes that some genes undergo rearrangement by tandem duplication followed by the random deletion of duplicated genes [58]. The translocation of tRNA genes in the *S. naso* mitogenome may be explained by TDRL. The gene block (*D*-*Y*-*G*-*M*) is repeated in tandem and thus produces two clusters of identical genes (*D*-*Y*-*G*-*M*)-(*D*′-*Y*′-*G*′-*M*′). Subsequently, redundant genes are likely randomly deleted. For example, *D*-*Y*-*G*-*M*-*D*′-*Y*′-*G*′-*M*′ (in which the underlined letters represent deleted genes) probably results in a new *M*-*D*′-*Y*′-*G*′ gene sequence.

Furthermore, *S. horrens*, *S. chloronotus*, *S. monotuberculatus*, and *S. naso* were found to have the same mitochondrial gene order, which was preliminarily identified as the ancestral gene order of *Stichopus* (Figure 4). It is possible that the ancestral gene order of *Stichopus* could be finally verified as more complete mitochondrial sequences in *Stichopus* are acquired. In addition, *Holothuria*, *Actinopyga*, *Parastichopus,* and *Apostichopus* species have the same gene order, while *Isostichopus* species have the same gene order as *Stichopus* species, which suggests a closer evolutionary relationship between *Isostichopus* and *Stichopus*.

### 3.5. Phylogenetic Analysis

**Figure 5:**
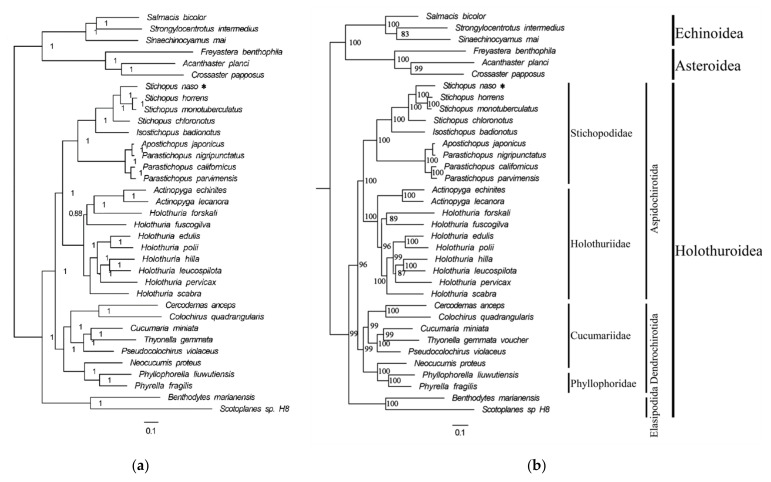
Phylogenetic trees constructed using the nucleotide sequences of the 13 PCGs in the *Stichopus naso* mitogenome. (**a**) BI method. (**b**) ML method. The numbers beside the nodes are posterior probabilities (BI) and bootstrap (ML), respectively.

The phylogenetic relationships were investigated on the basis of 13 PCGs from 28 Holothuroidea species, three Echinoidea species, and three Asteroidea species through ML and BI methods. The topologies of the phylogenetic trees determined by the BI and ML analyses are very similar on the whole (Figure 5). In the BI analysis, the genus *Holothuria* formed several clades, which were embedded by the genus *Actinopyga*. However, in the ML analysis, each family analyzed in Holothuroidea formed a separate clade, which was more consistent with their morphological classification. Nevertheless, the topology of Stichopodidae was consistent in both analyses.

In both phylogenetic trees, *S. horrens* and *S. monotuberculatus* formed a well-supported clade (BI posterior probabilities (PP) = 1; ML bootstrap (BP) = 100), which was consistent with the result of the phylogenetic tree constructed using the COI gene [43]. *Stichopus naso* was recovered as a sister to the *S. horrens*/*S. monotuberculatus* clade. Additionally, *S. chloronotus* and (*S. naso* + (*S. horrens* + *S. monotuberculatus*)) formed a monophyletic clade. This finding was consistent with previous phylogenetic studies, in which the phylogenetic trees were constructed based on the COI gene and the 16S gene, respectively [13,43,59]. Meanwhile, *I. badionotus* clustered with *Stichopus* with strong nodal support in the phylogenetic tree (Figure 5), which indicated a close relationship between *I. badionotus* and *Stichopus*; this cluster result was in line with those of previous studies [13,43,59,60]. In addition, both *I. badionotus* and *Stichopus* shared the identical rearrangement pattern of the genes in present study.

Holothuroids are a diverse group in echinoderms [60]. A broader analysis of the mitochondrial genomes in sea cucumbers would help to provide a more comprehensive understanding of their phylogenetic relationships. However, the available data on the mitochondrial genomes of sea cucumbers are limited. The complete mitochondrial genomes of more species need to be obtained and comprehensively studied to further elucidate the phylogenetic relationships of sea cucumbers and to identify confused species in sea cucumbers on a molecular level.

## 4. Conclusions

In this study, the complete mitochondrial genome of *S. naso* was characterized for the first time. The *S. naso* mitogenome is 16,239 bp in length and encodes 13 PCGs, 22 tRNA genes, and two rRNAs. The mitogenome composition exhibited positive AT-skews and negative GC-skews, which were consistent with most of the sequenced mtDNAs in Aspidochirotida. The gene order of *S. naso* is the same as those of other *Stichopus* species and *Isostichopus*, whereas it is different from those of other species of Aspidochirotida. The phylogenetic analysis based on the mitochondrial genomes of sea cucumbers demonstrated that *S. naso* belongs to a member of the Stichopodidae.

## Figures and Tables

**Table 1:** Summary of the genes in the *Stichopus naso* mitogenome.

Gene	Strand	Location	Size	Anticodon	Codon		Continuity
					Start	Stop	
*cox1*	+	1-1554	1554		ATG	TAA	167
*trnR*	+	1560-1627	68	TCG			5
*nad4l*	+	1628-1924	297		ATG	TAA	0
*cox2*	+	1925-2614	690		ATG	TAA	0
*trnK*	+	2635-2698	64	CTT			20
*atp8*	+	2699-2875	177		ATG	TAA	0
*atp6*	+	2869-3552	684		ATG	TAA	−7
*cox3*	+	3555-4337	783		ATG	TAA	2
*trnS2*	−	4336-4406	71	TGA			−2
*nad3*	+	4438-4782	345		GTG	TAG	31
*nad4*	+	4790-6157	1368		ATG	TAG	7
*trnH*	+	6148-6215	68	GTG			−10
*trnS1*	+	6218-6285	68	GCT			2
*nad5*	+	6286-8130	1845		ATG	TAA	0
*nad6*	−	8143-8631	489		ATG	TAA	12
*cob*	+	8640-9782	1143		ATG	TAA	8
*trnF*	+	9783-9853	71	GAA			0
*rrnS*	+	9852-10678	827				−2
*trnE*	+	10678-10746	69	TTC			−1
*trnT*	+	10749-10818	70	TGT			2
*trnP*	+	11483-11550	68	TGG			664
*trnQ*	−	11547-11616	70	TTG			−4
*trnN*	+	11626-11694	69	GTT			9
*trnL1*	+	11703-11774	72	TAG			8
*trnA*	−	11791-11858	68	TGC			16
*trnW*	+	11859-11928	70	TCA			0
*trnC*	+	11937-12002	66	GCA			8
*trnV*	−	12004-12075	72	TAC			1
*trnD*	−	12089-12156	68	GTC			13
*trnY*	+	12163-12230	68	GTA			6
*trnG*	+	12283-12351	69	TCC			52
*trnM*	+	12354-12422	69	CAT			2
*trnL2*	+	12436-12507	72	TAA			13
*nad1*	+	12508-13479	972		ATG	TAG	0
*trnI*	+	13487-13554	68	GAT			7
*nad2*	+	13555-14601	1047		GTG	TAA	0
*rrnL*	+	14577-16072	1496				−25

**Table 2:** Nucleotide composition of the whole *Stichopus naso* mitogenome and the genes.

Region	A%	T%	G%	C%	A + T (%)	G + C (%)	AT-Skew	GC-Skew
mitogenome	30.07	28.69	17.36	23.88	58.76	41.24	0.023	−0.158
PCGs	27.41	30.67	17.32	24.60	58.07	41.93	−0.056	−0.174
*cox1*	26.45	32.11	18.60	22.84	58.56	41.44	−0.097	−0.102
*nad4l*	26.26	37.71	12.79	23.23	63.97	36.03	−0.179	−0.290
*cox2*	28.84	30.00	17.10	24.06	58.84	41.16	−0.020	−0.169
*atp8*	33.90	29.38	15.25	21.47	63.28	36.72	0.071	−0.169
*atp6*	28.51	30.70	15.20	25.58	59.21	40.79	−0.037	−0.255
*cox3*	25.80	31.16	19.16	23.88	56.96	43.04	−0.094	−0.110
*nad3*	22.61	32.17	15.65	29.57	54.78	45.22	−0.175	−0.308
*nad4*	30.26	30.26	15.79	23.68	60.53	39.47	0.000	−0.200
*nad5*	31.65	26.67	16.42	25.26	58.32	41.68	0.085	−0.212
*nad6*	14.72	42.74	26.38	16.16	57.46	42.54	−0.488	0.240
*cob*	28.61	28.17	16.54	26.68	56.78	43.22	0.008	−0.235
*nad1*	23.56	30.86	19.34	26.23	54.42	45.58	−0.134	−0.151
*nad2*	26.17	30.75	16.14	26.93	56.92	43.08	−0.080	−0.251
tRNAs	32.94	29.05	19.83	18.18	61.99	38.01	0.063	0.043
rRNAs	35.13	23.25	20.06	21.57	58.37	41.63	0.203	−0.036

**Table 3:** The codon numbers and relative synonymous codon usages in 13 PCGs of *Stichopus naso* mitogenome.

Codon	Count	RSCU	Codon	Count	RSCU	Codon	Count	RSCU	Codon	Count	RSCU
UUU(F)	189	1.01	UCU(S)	138	1.65	UAU(Y)	78	0.98	UGU(C)	35	0.99
UUC(F)	186	0.99	UCC(S)	112	1.34	UAC(Y)	82	1.02	UGC(C)	36	1.01
UUA(L)	130	1.13	UCA(S)	80	0.95	UAA(*)	85	1.06	UGA(W)	75	1.20
UUG(L)	52	0.45	UCG(S)	40	0.48	UAG(*)	76	0.94	UGG(W)	50	0.80
CUU(L)	158	1.37	CCU(P)	107	1.37	CAU(H)	79	1.06	CGU(R)	26	0.89
CUC(L)	131	1.14	CCC(P)	88	1.13	CAC(H)	70	0.94	CGC(R)	19	0.65
CUA(L)	147	1.28	CCA(P)	87	1.12	CAA(Q)	105	1.13	CGA(R)	40	1.37
CUG(L)	73	0.63	CCG(P)	30	0.38	CAG(Q)	81	0.87	CGG(R)	32	1.09
AUU(I)	130	1.03	ACU(T)	100	1.14	AAU(N)	80	0.57	AGU(S)	48	0.57
AUC(I)	91	0.72	ACC(T)	102	1.17	AAC(N)	96	0.69	AGC(S)	67	0.80
AUA(I)	157	1.25	ACA(T)	97	1.11	AAA(N)	242	1.74	AGA(S)	124	1.48
AUG(M)	95	1.00	ACG(T)	51	0.58	AAG(K)	128	1.00	AGG(S)	62	0.74
GUU(V)	69	1.19	GCU(A)	85	1.31	GAU(D)	60	0.94	GGU(G)	63	1.09
GUC(V)	51	0.88	GCC(A)	84	1.29	GAC(D)	67	1.06	GGC(G)	31	0.53
GUA(V)	73	1.26	GCA(A)	60	0.92	GAA(E)	117	1.33	GGA(G)	89	1.53
GUG(V)	38	0.66	GCG(A)	31	0.48	GAG(E)	59	0.67	GGG(G)	49	0.84

## Data Availability

The mitochondrial genome was deposited at NCBI, with accession number MZ469138.

## Supplementary Materials

The following are available online at https://www.mdpi.com/article/10.3390/genes13050825/s1. Table S1: GenBank accession numbers, base compositions, and skew values of the species analyzed in this paper.
