# Supplementary material for: The Complete Mitochondrial Genome of Stichopus naso (Aspidochirotida: Stichopodidae: Stichopus) and Its Phylogenetic Position
Source: Genes (Basel). 2022 May 5;13(5):825. doi: 10.3390/genes13050825 (PMC9141342; doi:10.3390/genes13050825)
Supplement: Supplementary file 1 [file genes-13-00825-s001.zip › genes-1694868-supplementary.pdf]

Supplement Table S1 GenBank accession numbers, base compositions and skew values of the species analyzed in this paper.

Species	GenBank No	A+T%	AT-skew	GC-skew
Holothuria polii	LR694133.1	58.33	0.060	-0.227
Holothuria leucospilota	NC_046849.1	57.55	0.103	-0.220
Holothuria fuscogilva	MZ305460.1	59.00	0.140	-0.176
Holothuria hilla	MN163001.1	59.63	0.088	-0.206
Holothuria edulis	NC_051928.1	58.86	0.097	-0.258
Holothuria pervicax	NC_045853.1	59.80	0.073	-0.174
Holothuria scabra	NC_027086.1	59.74	0.094	-0.209
Holothuria forskali	NC_013884.1	62.22	0.009	-0.134
Actinopyga echinites	MN793975.1	62.94	0.083	-0.129
Actinopyga lecanora	MW248463.1	63.40	0.093	-0.173
Stichopus monotuberculatus	NC_052743.1	60.38	0.027	-0.192
Stichopus chloronotus strain lv	NC_056131.1	58.55	0.075	-0.225
Stichopus horrens	NC_014454.1	60.11	0.025	-0.189
Parastichopus nigripunctatus	NC_013432.1	61.82	0.025	-0.055
Parastichopus parvimensis	NC_029699.1	61.69	0.030	-0.064
Parastichopus californicus	NC_026727.1	61.40	0.027	-0.066
Isostichopus badionotus	MZ188901.1	60.78	0.039	-0.196
Apostichopus japonicus	NC_012616.1	61.96	0.026	-0.061
Benthodytes marianensis	NC_040968.1	69.24	-0.066	-0.160
Scotoplanes sp. H8	LC416626.1	72.56	-0.060	-0.199
Phyllophorella liuwutiensis	NC_057437.1	65.20	0.093	-0.273
Phyrella fragilis	MZ305459.1	64.32	0.133	-0.313
Thyonella gemmata voucher	MZ463652.1	64.37	0.122	-0.301
Neocucumis proteus	MZ305458.1	63.51	0.147	-0.294
Cercodemas anceps	NC_054245.1	69.60	0.203	-0.233
Pseudocolochirus violaceus	NC_051967	63.61	0.216	-0.336
Colochirus quadrangularis	NC_051929.1	66.93	0.202	-0.316
Cucumaria miniata	NC_005929.1	63.83	0.120	-0.269
Salmacis bicolor	KU302104.1	60.27	0.133	-0.205
Sinaechinocyamus mai	MN103227.1	62.96	-0.023	-0.166
Strongylocentrotus intermedius	KC490912.1	58.92	-0.031	-0.104
Acanthaster planci	NC_007788.1	56.34	0.104	-0.280
Crossaster papposus	NC_053361.1	67.21	0.056	-0.227
Freyastera benthophila	NC_039982.1	68.23	0.017	-0.330
Stichopus monotuberculatus mt rRNAs	-	59.93	0.183	-0.052
Stichopus chloronotus strain lv mt rRNAs	-	58.71	0.230	-0.060
Stichopus horrens mt rRNAs	-	60.15	0.192	-0.066
